# Pharmacological Privilege: How Glucagon-Like Peptide-1 (GLP-1) Medications are Widening Health Inequalities

**DOI:** 10.7759/cureus.97124

**Published:** 2025-11-17

**Authors:** Ailie H Brennan-Davies, Samuel Lakey

**Affiliations:** 1 Internal Medicine, St Vincent’s Hospital, Melbourne, AUS; 2 Cardiology, Northumbria Healthcare NHS Foundation Trust, Newcastle Upon Tyne, GBR

**Keywords:** glp-1 agonist, health inequalities, medical weight loss, obesity treatment, public health policy

## Abstract

Obesity remains a growing cause of morbidity and mortality, disproportionately affecting socioeconomically disadvantaged groups. Glucagon-like peptide-1 (GLP-1) agonist medications promote significant weight loss, giving hope to public healthcare systems increasingly stretched by obesity-related cardiometabolic disorders. Despite increased use of GLP-1 agonists for this indication, the need for these medications greatly outstrips supply due to financial and logistical constraints. The high cost of these medications is currently prohibitive to widespread rollout, yet they remain accessible privately for those able to afford them. This disparity reflects Julian Tudor Hart’s inverse care law, in which access to effective healthcare inversely correlates with need. Achieving fair and sustainable integration of GLP-1 therapies will require evidence-informed policies that ensure treatment based on need rather than means. Equitable global access, supported by investment in preventive, population-level strategies, is essential to promote health and avoid deepening worldwide health inequalities.

## Editorial

The global obesity epidemic, which itself was accelerated by the COVID-19 pandemic, is an increasing cause of morbidity and mortality and a subsequent drain on healthcare resources in the United Kingdom and worldwide [1]. Obesity is a multi-system disease with numerous anatomical and metabolic consequences that increase the risk of cardiovascular disease, type 2 diabetes, musculoskeletal disorders, and certain cancers [2]. This snowballing effect of associated comorbidities directly increases healthcare costs through higher rates of outpatient visits, medication use, and longer hospital admissions [3]. Its hidden costs are also significant, including economic losses from reduced productivity and unemployment, as well as personal costs from a diminished quality of life due to medical complications, mental health challenges, and social stigma [4,5]. Increasingly, individuals are developing obesity at younger ages, leading to prolonged exposure to these morbidities and an accumulation of these costs [6].

The rise of glucagon-like peptide-1 (GLP-1) receptor agonists has been hailed as a breakthrough in the fight against obesity. Originally developed for type 2 diabetes, these medications have demonstrated remarkable efficacy in promoting weight loss and reducing obesity-related complications such as heart attacks and strokes [7]. Yet, beneath the surface of this exciting pharmacological breakthrough lies the concern that these drugs are leading to widening healthcare inequalities, both within the UK National Health Service (NHS) and globally.

Dr. Julian Tudor Hart stated the following in his seminal work published, now over half a century ago, in The Lancet: “The availability of good medical care tends to vary inversely with the need for it in the population served. This inverse care law operates more completely where medical care is most exposed to market forces, and less so where such exposure is reduced” [8]. GLP-1 agonists perfectly illustrate this dynamic. It is a well-established fact that the populations with the highest prevalence of obesity and its complications are disproportionately socioeconomically disadvantaged [9]. At present, only a minority of these people can access these costly medications at subsidised rates through public healthcare, while an explosion in private clinics and prescriptions provides a rapid route to GLP-1 agonist treatment for people with disposable income. This divide enables wealthier individuals to access weight loss and metabolic risk reduction that poorer patients cannot obtain [10].

In response to the significant long-term costs of managing multimorbidity in obese patients, the UK’s National Institute for Health and Care Excellence (NICE) recommends GLP-1 agonists as a cost-effective treatment for those with established weight-related conditions [11]. However, cost-effectiveness does not guarantee affordability. GLP-1 agonists remain under patent protection [12], making them prohibitively expensive for widespread use within the NHS, where finite resources must be balanced across competing healthcare demands. Wider adoption also requires significant upfront investment in administration infrastructure. Until early 2025, GLP-1 medications were restricted to specialist NHS obesity services, which had limited capacity and long waiting lists. New guidelines allow tirzepatide, a dual glucose-dependent insulinotropic polypeptide (GIP) and GLP-1 agonist, to be prescribed in primary care, thereby expanding patient access [11]. Despite NICE guidelines recommending tirzepatide for an estimated 3.4 million people with a body mass index (BMI) >35 kg/m² and at least one related complication, the initial rollout is highly restricted. Currently, only patients with a BMI >40 kg/m² and four or more comorbidities, fewer than 250,000 individuals, are eligible, targeting only those at the highest risk [13]. Furthermore, the NHS “postcode lottery,” in which local commissioning decisions lead to regional differences in care, is likely to exacerbate inequalities before private access is even considered.

The NHS currently limits GLP-1 agonist prescriptions for obesity to a maximum of two years, yet research suggests that sustained benefit requires long-term therapy. Evidence of weight regain after cessation raises concerns that patients may be reliant on these expensive medications lifelong, with only those who can afford ongoing treatment maintaining improvements in health outcomes [14]. We risk a pattern developing in which patients taking GLP-1 agonists lose weight during treatment, then lose access after two years of NHS coverage or when they can no longer afford private prescriptions, leading to weight regain. This “yo-yo” effect, known as weight cycling, is associated with increased cardiometabolic risks, independent of high baseline BMI, including dyslipidemia, hyperglycemia, and hypertension [15]. Lack of sustained weight loss on cessation is likely to be demoralizing for patients and may reduce motivation for future attempts to achieve a healthy weight. For patients taking GLP-1 agonists for diabetes management, lower income is associated with higher discontinuation rates [16]. If this trend persists as GLP-1 agonists are widely adopted for obesity, policies will need to address barriers to sustained use to avoid widening disparities in long-term outcomes among disadvantaged groups.

Despite the NHS’s tightly controlled criteria for GLP-1 agonists, UK patients have a significant advantage. Many comparable high-income countries, including Germany, Italy, and Denmark, offer no public reimbursement for these medications for weight loss, making them accessible only to those able to afford out-of-pocket payment [17]. Similarly, the nationwide Medicare program in the United States does not cover pharmacological treatments for obesity, and only a minority of states (13 out of 50) provide limited coverage through Medicaid programs for a small subset of patients [18]. Disparities in access are particularly stark in the United States, where medications are often substantially more expensive and patients are more reliant on private insurance than in other high-income countries. Owing to the lack of standardized global pricing agreements, European countries benefit from lower GLP-1 agonist costs. A month’s supply of semaglutide costs approximately US$93 in the United Kingdom and as little as US$51 in Croatia, compared to US$936 in the United States [19]. Consequently, without comprehensive health insurance, which is often a privilege of higher-income individuals, significant disposable income is required to afford these medications in the United States.

Medication shortages create a dual injustice. The 2023-2024 shortfall of semaglutide, exacerbated by off-label demand from affluent populations, critically reduced access to a vulnerable patient group: those with type 2 diabetes, who are often from lower-income and minority backgrounds and rely on these medications for essential glycemic control [20]. Shortages have increased the popularity of compounded GLP-1 agonists (pharmacy-made, customized medications), with significant efficacy and safety concerns. These unregulated products are associated with greater medication errors, drug contaminations, adverse effects, and hospitalizations [21]. Being less expensive, compounded GLP-1 agonists are more accessible to lower-income patients than branded versions, thereby disproportionately increasing their exposure to related harms.

These inequities are mirrored on a global stage. Obesity is rising most rapidly in low- and middle-income countries, particularly across Asia and Africa, driven by urbanization, dietary shifts toward processed food, and reduced physical activity [22]. These nations face significant unmet health needs and lack the financial capacity for high-cost medications such as GLP-1 agonists. This reflects an established pattern in which new treatments are first accessible to wealthy countries, a trend seen with AIDS antiretrovirals, COVID-19 vaccines, and cancer therapies [23-25]. The World Health Organization has issued warnings about counterfeit GLP-1 agonists associated with serious potential harms [26]. This poses a particular risk to low-income countries, where weaker regulatory systems and fragmented supply chains increase vulnerability to falsified medications [27]. Without safe and equitable access to GLP-1 agonists, through global licensing or tiered access schemes, the rising obesity burden will disproportionately affect low-income countries, further deepening global health disparities.

In addition to the financial costs, there are other harms associated with GLP-1 agonists that may further widen health inequalities. The rapid weight loss induced by these medications also reduces skeletal muscle mass, a key metabolic organ, necessitating concurrent resistance training and a high-protein diet. This represents a disproportionate challenge for lower socioeconomic groups facing financial and practical barriers to both protein-dense nutrition and exercise [28,29]. GLP-1 agonist-assisted weight loss without integrated support for muscle preservation may accelerate frailty and sarcopenia, disproportionately affecting those without access to multidisciplinary care [30]. These harms will be most immediately apparent among the frail and elderly; however, with obesity being an illness of all ages, there is a risk of these drugs making frailty a pathology of all ages. Other well-documented side effects are primarily gastrointestinal, though serious complications such as pancreatitis and gallbladder disease can occur. While widely considered safe, GLP-1 agonists are relatively novel medications globally, first approved for diabetes management in the United States in 2005. Approval for weight loss came nearly a decade later, and therefore, long-term data on tolerability, efficacy, and side effects remain limited, necessitating ongoing safety monitoring.

Obesity and its associated comorbidities can both directly and indirectly limit earning potential and career advancement through loss of productivity and job discrimination [31]. This bidirectional relationship between obesity and financial income reinforces a feedback loop in which the wealthy can both afford GLP-1 agonists and enjoy enhanced economic opportunities from their use.

Ultimately, where access to medications is mediated by the ability to pay, availability concentrates among the affluent, exactly as Dr. Tudor Hart predicted. This inequality in access risks creating a society where obesity is a disease solely of the economically disadvantaged, increasing stigma and social discrimination.

Headline-grabbing press releases associated with GLP-1 agonists have penetrated the psyche of the nation and further reinforce the idea that pharmacotherapy is the best solution to societal problems such as obesity. The medicalization of obesity with a “quick fix” solution risks disincentivizing society from proven lifestyle improvement measures, despite added health and psychological benefits beyond weight management. Marketing GLP-1 agonists as a “cure” for obesity naturally detracts from the importance of prevention and leaves the fundamental drivers of the epidemic unaddressed. This risks diverting public resources and political energy away from upstream interventions that may reduce obesity prevalence more equitably across the whole population, such as regulations on ultra-processed foods and urban planning that encourage active travel.

GLP-1 agonists exemplify the inverse care law because they concentrate powerful clinical benefits among the affluent, while leaving those in greater need with limited access. The introduction of GLP-1 agonists has changed the face of obesity management at a time of crucial need, with increasing prevalence and related morbidity. The expanded use of these medications, broadly deemed safe and cost-effective, has the potential to improve outcomes for thousands in the United Kingdom alone. Introducing cheaper generic GLP-1 agonists could help cap prices and expand access, particularly for lower-income populations; however, this would require existing patents to expire or voluntary licensing agreements from manufacturers. Until then, without deliberate policy interventions to ensure equitable access and integrated multidisciplinary care, these drugs risk deepening national and global health inequities in the way Dr. Tudor Hart warned over 50 years ago.
